# The BACH1 inhibitor ASP8731 inhibits inflammation and vaso-occlusion and induces fetal hemoglobin in sickle cell disease

**DOI:** 10.3389/fmed.2023.1101501

**Published:** 2023-04-18

**Authors:** John D. Belcher, Selvaraj Nataraja, Fuad Abdulla, Ping Zhang, Chunsheng Chen, Julia Nguyen, Conglin Ruan, Maneet Singh, Shilpa Demes, Lyndsay Olson, Domi Stickens, Jeff Stanwix, Emer Clarke, Yongzhao Huang, Margaret Biddle, Gregory M. Vercellotti

**Affiliations:** ^1^Department of Medicine, Division of Hematology, Oncology and Transplantation, University of Minnesota, Minneapolis, MN, United States; ^2^Mitobridge Inc., Cambridge, MA, United States; ^3^Astellas Pharma Global Development Inc., Northbrook, IL, United States; ^4^ReachBio, Seattle, WA, United States

**Keywords:** BACH1, Nrf2, sickle cell disease, hemoglobin F, gamma globin, HMOX1, vaso-occlusion, NF-kappa B

## Abstract

In sickle cell disease (SCD), heme released during intravascular hemolysis promotes oxidative stress, inflammation, and vaso-occlusion. Conversely, free heme can also activate expression of antioxidant and globin genes. Heme binds to the transcription factor BACH1, which represses NRF2-mediated gene transcription. ASP8731, is a selective small molecule inhibitor of BACH1. We investigated the ability of ASP8731 to modulate pathways involved in SCD pathophysiology. In HepG2 liver cells, ASP8731 increased *HMOX1* and *FTH1* mRNA. In pulmonary endothelial cells, ASP8731 decreased *VCAM1* mRNA in response to TNF-α and blocked a decrease in glutathione in response to hemin. Townes-SS mice were gavaged once per day for 4 weeks with ASP8731, hydroxyurea (HU) or vehicle. Both ASP8731 and HU inhibited heme-mediated microvascular stasis and in combination, ASP8731 significantly reduced microvascular stasis compared to HU alone. In Townes-SS mice, ASP8731 and HU markedly increased heme oxygenase-1 and decreased hepatic ICAM-1, NF-kB phospho-p65 protein expression in the liver, and white blood cell counts. In addition, ASP8731 increased gamma-globin expression and HbF+ cells (F-cells) as compared to vehicle-treated mice. In human erythroid differentiated CD34+ cells, ASP8731 increased *HGB* mRNA and increased the percentage of F-cells 2-fold in manner similar to HU. ASP8731 and HU when given together induced more HbF+ cells compared to either drug alone. In CD34+ cells from one donor that was non-responsive to HU, ASP8731 induced HbF+ cells ~2-fold. ASP8731 and HU also increased *HBG* and *HBA*, but not *HBB* mRNA in erythroid differentiated CD34+ cells derived from SCD patients. These data indicate that BACH1 may offer a new therapeutic target to treat SCD.

## Introduction

Sickle cell disease (SCD) is caused by a point mutation in the β-globin chain of hemoglobin, resulting in a valine substitution for glutamate at position 6 resulting in the mutation of hemoglobin A (HbA, α_2_/β_2_) to hemoglobin S (HbS, α_2_/β^S^_2_). When deoxygenated during transit through the venous circulation, HbS polymerizes, which leads to the formation of red blood cells (RBCs) that are dehydrated, stiffer, more adhesive, and abnormally shaped (1). Repeated bouts of HbS polymerization and depolymerization as RBCs circulate through the venous and arterial vasculature shortens their lifespan and promotes intravascular and extravascular hemolysis that releases toxic free heme. Free heme promotes oxidative stress, inflammation, and vaso-occlusion (2, 3).

Despite its toxicity, free heme also activates antioxidant and globin gene expression (4–9). Heme binds to BTB and CNC homolog 1 (BACH1), which functions as a transcriptional repressor of nuclear factor erythroid 2-related factor 2 (NRF2) (10). The release of heme-BACH1 from antioxidant response elements (ARE) permits the binding of NRF2 to ARE and the cell-specific transcription of antioxidant genes such as *HMOX1* (heme oxygenase 1, HO-1), *GR* (glutathione reductase), and *NQO1* (NAD(P)H dehydrogenase [quinone] 1). Increasing HO-1 expression has been shown to reduce inflammation, adhesion molecules, and stasis in a mouse model of SCD (11, 12). Additionally, polymorphisms in the *HMOX1* promoter that correspond to increased HO-1 levels have been correlated with reduced incidents of acute chest syndrome in pediatric SCD patients and overall reductions in vaso-occlusive pain crises and hospitalization rates (13, 14).

Previous studies demonstrated increases in nuclear Nrf2 expression and downstream expression of Nrf2-responsive genes including *Hmox1*, *Nqo1*, and *Hbg* (gamma globin) after administration of dimethyl fumarate (DMF) to murine SCD models (15, 16). Importantly, heme-induced microvascular stasis was inhibited in an HO-1 dependent manner (15). In addition, DMF increased the expression of other NRF2-responsive genes including proteins involved in hemoglobin, heme, and iron clearance as well as a decrease in markers of inflammation such as nuclear factor kappa B (NF-ĸB) phospho-p65, toll-like receptor 4 (TLR4), adhesion molecules, and pro-inflammatory cytokines (3, 15, 16). A decrease in hepatic lesions and increased circulating hemoglobin F (HbF)-containing RBCs (F-cells) was also observed in these studies (3, 15, 16). Conversely, a loss of NRF2 function exacerbates SCD pathophysiology and inhibits HbF expression (17, 18).

ASP8731 (previously known as ML-0207) was identified as a selective small molecule BACH1 inhibitor that relieves BACH1 repression of NRF2 pathways in human and murine cells (19). We investigated the capability of ASP8731 to increase antioxidant and anti-inflammatory gene expression in cell culture, decrease microvascular stasis (vaso-occlusion) and white blood cell (WBC) counts, and induce gamma globin and F-cells in a preclinical murine model of SCD and human CD34 cells during erythroid differentiation.

## Materials and methods

### Mice

Animal experiments were approved by the Institutional Animal Care and Use Committee at the University of Minnesota. These studies used male and female knock-in Townes-sickle (HbSS) mice (hα/hα, hγ^A^/hγ^A^, hβ^S^/hβ^S^) on a 129/B6 mixed genetic background (20). All mice were genotyped, housed in specific pathogen-free rooms to limit infections, and kept on a 12-h (h) light/dark cycle at 21°C. All animals were monitored daily for health problems, food and water levels, and cage conditions. All animals were included in each analysis and there were no adverse events that required changes to the protocol. Mice were 12–16 weeks of age.

### ASP8731 and hydroxyurea administration to mice

ASP8731 is a selective small molecule BACH1 inhibitor, which activates antioxidant NRF2-responsive genes (19). We examined the cytoprotective effects of ASP8731 on human cells and in Townes HbSS mice. Mice were gavaged (10 ml/kg) once daily for 14 days consecutively or 6 days per week for 4 weeks with the indicated dose of ASP8731 or hydroxyurea (HU) suspended in 0.05% w/v Tween 80 + 0.45% hydroxypropyl methylcellulose (HPMC).

### Tissue collection

At the end of each experiment, mice were euthanized in a CO_2_ atmosphere and blood was collected from the inferior vena cava into EDTA tubes and processed at 4°C. Livers were excised, flash frozen in liquid N_2_, and stored at -85°C until use.

### HepG2 cell culture and gene expression

Human hepatoma cells (HepG2, ATCC HB-8065) were grown in Minimum Essential Media (EMEM Corning #MT-10-010-CV) supplemented with 1X MEM Non-essential Amino Acids Solution (Gibco 100X Solution #11140–50), 1 mM sodium pyruvate (Gibco 100 mM solution, #MT-25-000-CI) and 10% heat-inactivated fetal bovine serum (Gibco #16140–071). Cells were spilt 2–3 times weekly at 1:4 or 1:6. HepG2 cells were plated at a density of 250 k cells in 12-well plates coated with collagen (ENZO Life Sciences, Cat#ALX-522-440-0050). Approximately 24 h post-seeding, cells were treated with a dose titration of ASP8731 or DMF (Fisher Scientific Cat#50–144-5120) as a positive control *via* media change at three biological replicates per treatment. Compounds were dissolved in 0.1% DMSO, untreated wells had the same concentration of DMSO to account for any effect of the vehicle. At 24 h post-treatment, cells were washed in PBS and frozen (-80°C) before RNA isolation and analysis as described below. Further, in independent experiment, cells were checked for treatment toxicity by MTT assay (no toxicity observed- data not shown).

### PAEC cell culture and gene expression

Human primary pulmonary arterial endothelial cells (PAEC, Lonza CC-2530) were grown in EGM-2 media (Lonza CC-3162). Cells were sub-cultured when they reached 70–85% confluence. Cells were seeded at 5 K viable cells/cm^2^. HPAEC were plated at 200 K/cells per well on 12-well plates (TrueLine #TR5001) in growth media and cultured at 37°C in 5% CO_2_. Approximately 24 h post-seeding, cells were treated with dose titration of ASP8731 in 0.1% DMSO *via* media change, control cells had the same amount of DMSO (*n* = 3 replicates per treatment). At 24 h post-treatment with ASP8731, TNF-α (10 ng/ml, Invitrogen) or hemin (50 μM, Sigma-Aldrich) was added to the media and the cells were incubated an additional 4 h (TNF-α) or 30 min (hemin). Treatment of 50 uM hemin for 30 min was determined to be the minimal dose that elicits a decrease in GSH, and is within the physiological levels reported in SCD and thalassemia patients. After treatment was complete, media was aspirated off, cells were washed with PBS and aspirated dry. For the TNF-α experiments, RNA was isolated and *VCAM1* mRNA was measured by Nanostring gene expression analysis as described below. For the hemin experiments, cellular glutathione (GSH) was measured using a GSH-Glo™ glutathione assay kit (Promega) following the manufacturer’s protocol. Cell viability was observed by MTT assay as described by the manufacturer (Sigma-Aldrich, Cat# CT02).

### CD34 cell isolation, expansion, and differentiation

Six normal human bone marrow (BM) samples from 2 African Americans, 3 Hispanics, and 1 Caucasian were obtained from three commercial sources (Lonza, NorCal Biologics, and iSpecimen). SCD subject whole blood (~50 ml/subject) was obtained from Sanguine Biosciences (*n* = 3 subjects). The whole blood was diluted 2-fold with PBS containing 2% FBS and then layered on ficoll for peripheral blood mononuclear blood cell (PBMC) isolation using density gradient centrifugation. The whole blood was centrifuged at 1200 rpm for 25 min and PBMC were harvested and counted. PBMC and BM derived CD34+ cells were enriched using Miltenyi’s CD34+ microbeads (positive selection method). BM derived CD34^+^ cells were stored in liquid nitrogen (−152°C) until required for HbF induction. On the day of the experiment, CD34+ cells were thawed in Iscove’s modified Dulbecco’s medium (IMDM, Hyclone) containing 10% heat-inactivated fetal bovine serum (FBS) and then cells were pelleted by centrifugation. The cell pellets were re-suspended in X-Vivo medium (Lonza) and viability was assessed with trypan blue. CD34^+^ cells from SCD whole blood were cultured soon after isolation. CD34^+^ cells were expanded in X-Vivo media containing rhIL-3 (10 ng/ml), rhSCF (100 ng/mL) and rhFlt-3 ligand (100 ng/mL). Approximately 10,000–12,500 cells in 250 μL volume were plated per well in round-bottom 96-well plates and culture for 7 days in a humidified incubator at 37°C/5% CO_2_. After 7 days, the cells were washed twice with X-Vivo media and resuspended in freshly prepared erythroid differentiation media containing X-Vivo-15, rhIL-3 (10 ng/mL), rhSCF (100 ng/mL), and Epo (3 U/mL). BACH1 inhibitor ASP8731 (1, 3, 10 μM) and/or HU (3, 10 μM) positive control for HbF induction were added either alone or in combination to the media. Standard cultures (solvent and compound free) and solvent control cultures (containing DMSO) were also initiated. Each test condition was set up in triplicate. Following culture in differentiation media for 7 days, the induction of HbF was assessed by flow cytometry. In addition, cells were collected on days 3 or 7 of differentiation for gene expression analysis. RNA from erythroid differentiated CD34 cells was isolated and analyzed for gene expression as described below.

### mRNA analysis

RNA was isolated using Machery-Nagel isolation kit (NucleoSpin RNA, Cat#740955.250) and quantified using a NanoDrop spectrophotometer (ThermoFisher Scientific). Isolated RNA was analyzed on a NanoString SPRINT profiler and nSolver version 4.0 software. A custom Tag-24 CodeSet and exploratory gene expression biomarker probes, were hybridized with a total mRNA input of 200 ng. To hybridize the probes to the target genes of interest, a thermocycler block was set to 67C (with the lid set to 72C) and samples were incubated overnight for a total of 16 h. The samples were cooled to 4C at the end of 16 h and remained at 4C until samples were pooled and added to wells of a SPRINT cartridge. Positive and negative controls were assessed for run QC, ensuring linearity and ample assay limit of detection, respectively. Raw data from the reference genes in study samples were assessed for stability across the experiment utilizing the GeNorm protocol for assessment of expression. Reference genes that exhibited differential expression ie oscillated randomly or excessively above/below the experiment geometric mean were excluded from content normalization calculations. The limit of detection for each sample was determined as the average of negative controls in each sample plus two times the standard deviation of the negative controls within the sample; the experimental limit of detection was calculated as the geometric mean of all individual limit of detection calculations across the experiment. CLTC, POLR2A, RPL27, and TBP were used as reference genes for normalization. Data was graphed as fold-change compared to DMSO treatment. Probe Sequences are listed in Supplementary Table 1.

### Measurement of microvascular stasis (vaso-occlusion)

After 4 weeks treatment with ASP8731 or HU, HbSS mice were anesthetized with ketamine and xylazine and dorsal skinfold chambers were implanted (21). Flowing subcutaneous venules (20–23 venules/mouse) in the chamber window were selected and mapped using intravital microscopy as previously described (21). After selection of flowing venules, mice were infused with a bolus infusion *via* tail vein with hemin (3.2 μmol/kg). One hour after hemin infusion, the venules were visually re-examined for stasis (no flow). The static venules in each mouse were counted and percent stasis at 1 h was calculated by dividing the number of static venules by the total (static + flowing) number of venules.

### Western blots

Hepatic microsomes and nuclear extracts were isolated from frozen organs as previously described (12). Immunoblots of cellular subfractions (30–50 μg protein) were run on 4–20% SDS PAGE gels (Bio-Rad #3450033), transferred to PVDF membranes, and immunostained with primary antibodies to NF-ĸB phospho-p65 (Ser536, Cell Signaling #3031), total p65 (Cell Signaling #3034), ICAM-1 (Abcam #ab124759), HO-1 (Enzo #ADI-111), BACH1 (US Biological Life Sciences #220980), or GAPDH (Sigma-Aldrich #G9545). Primary antibodies were detected with suitable secondary antibodies conjugated to alkaline phosphatase and visualized with ECF substrate (GE Healthcare) and a Typhoon FLA 9500 imager (GE Healthcare).

### White blood cell (WBC) counts

EDTA blood was diluted 1:20 or 1:40 in 3% glacial acetic acid to lyse RBCs. WBCs were counted manually using a hemocytometer and a light microscope.

### HbF by FACS

Differentiated CD34+ cells collected at day 7 of erythroid differentiation were washed with PBS. For flow cytometry analysis, non-specific binding of antibodies was blocked with 10% FBS with human IgG (20 μg/mL) at 4°C for 20 min. Following blocking, the surface markers of the cells were stained with an antibody cocktail containing CD235a-PE/Cy7, and CD71-APC at 4°C for 30 min. The cells were washed with PBS, re-suspended in 1x Cytofix/Cytoperm solution (BD Biosciences), and fixed for 20 min at 4°C. Following fixation, cells were washed with Perm/Wash Buffer (BD Biosciences) and stained with anti-HbF-PE for 30 min at room temperature. For isotype control, IgG-PE was used in place of HbF-PE. Finally, the cells were washed twice with Perm/Wash Buffer, resuspended in PBS containing 2% FBS, and then analyzed by flow cytometry using a Beckman Coulter CytoFlex cytometer. Both percentages of various cell populations as well as events (cells) per μL were collected. The HbF^+^ cells were found within the CD71^+^ bright cell population. HU was used as a positive control throughout the experiment.

### Statistical analysis

Comparisons of multiple treatment groups were made using one-way ANOVA with Tukey’s or Dunnett’s multiple comparison test (GraphPad Prism version 9.4). Normality of data was assessed using the Shapiro–Wilk test. Non-normally distributed data was analyzed for differences between treatment groups using the Kruskal-Wallis test with Dunn’s multiple comparisons test.

## Results

### Effects of ASP8731 on antioxidant and anti-inflammatory genes *in vitro*

**Figure 1:**
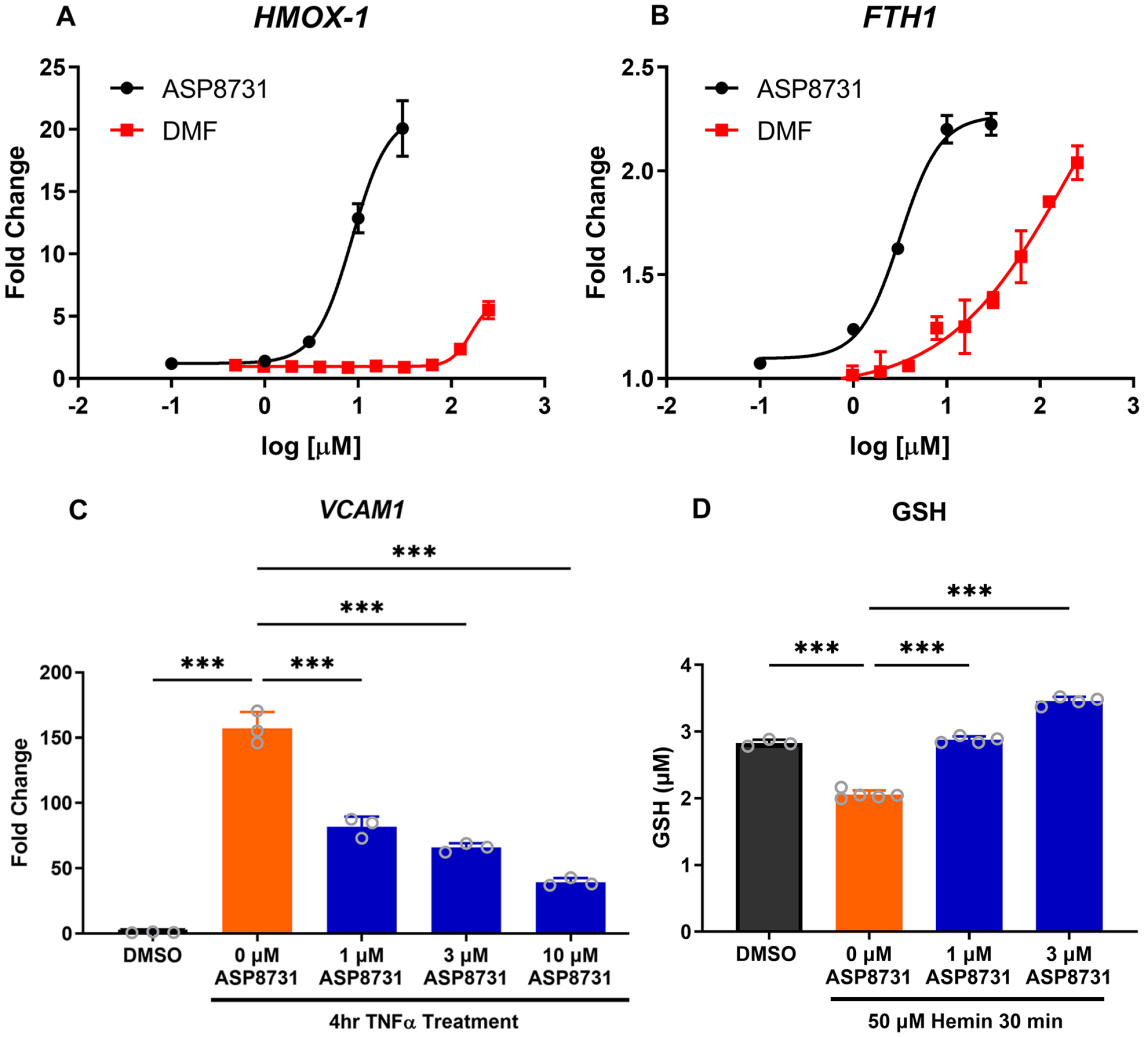
BACH1 inhibitor ASP8731 modulated multiple pathways *in vitro* that are involved in SCD pathophysiology. ASP8731, dimethyl fumarate (DMF), or DMSO vehicle were incubated with HepG2 cells for 24 h and **(A)** *HMOX1* (HO-1) and **(B)** *FTH* (ferritin heavy chain) mRNA were measured. **(C)** Human primary pulmonary arterial endothelial cells (PAEC) were incubated with ASP8731 (1, 3, 10 μM) or DMSO vehicle for 24 h and then TNF-α (10 ng/ml), was added for an additional 4 h followed by RNA isolation and measurement of *VCAM1* mRNA. **(D)** PAEC were incubated with DMSO vehicle or ASP8731 (0, 1, or 3 μM) for 24 h and then hemin (50 μM) was added for an additional 30 min followed by measurement of glutathione (GTH) levels. Values are means ± SEM. ***p < 0.001, one-way ANOVA with Tukey’s multiple comparison test.

Since BACH1 represses NRF2-responsive pathways in cells, we investigated the ability of ASP8731 to modulate antioxidant and anti-inflammatory genes in cell culture. HEPG2 cells were treated with increasing concentrations of the BACH1 inhibitor ASP8731 (0.1–50 μM) or DMF (1–250 μM), a positive control for NRF2 gene transcription, for 24 h. ASP8731 potently induced HO-1 (*HMOX1*) and ferritin heavy chain (*FTH*) mRNA in HEPG2 cells, even more potently than DMF (Figures 1A,B). Human primary pulmonary arterial endothelial cells (PAEC) were incubated with ASP8731 (0, 1, 3, 10 μM) or DMSO vehicle for 24 h and then TNF-α (10 ng/ml), was added for an additional 4 h followed by RNA isolation and measurement of *VCAM1* mRNA. *VCAM1* mRNA was maximally increased in TNF-α-treated cells in the absence of BACH1 inhibitor ASP8731 and decreased as the concentration of ASP8731 increased (Figure 1C). PAEC were incubated with DMSO vehicle or ASP8731 (0, 1, or 3 μM) for 24 h and then cells were exposed to heme-mediated oxidative stress. Hemin (50 μM) was added for an additional 30 min followed by measurement of glutathione (GSH) levels as a marker of oxidative stress. GSH levels were decreased in hemin-treated cells in the absence of BACH1 inhibitor ASP8731 and increased as the concentration of ASP8731 increased (Figure 1D). The effects of treatments on HepG2 and PAEC viability were measured by MTT assay. ASP8731 at the doses tested did not affect the viability of HepG2 cells or HPAEC cells in the presence of DMSO or hemin.

### Microvascular stasis (vaso-occlusion) in HbSS mice

**Figure 2:**
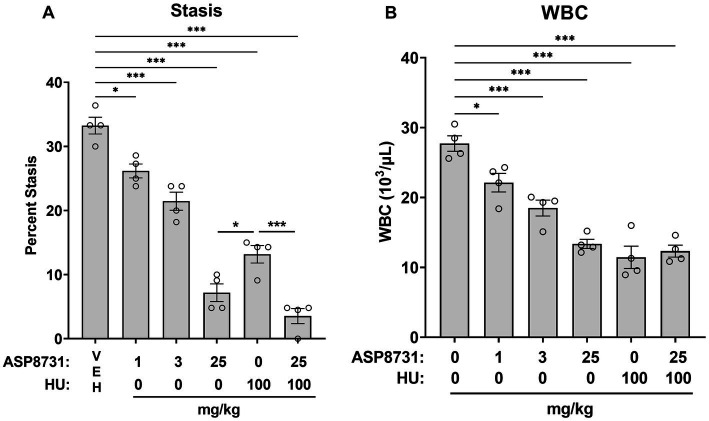
Bach1 inhibitor ASP8731 decreases microvascular stasis and WBC counts in Townes HbSS mice. Townes HbSS mice were gavaged once daily, 6 days per week for 4 weeks with vehicle (VEH), ASP8731, and/or HU at the indicated doses (n = 4 mice/group, 2 male and 2 female). On the last day of treatment, mice were implanted with dorsal skinfold chambers and infused with hemin (3.2 μmol/kg body weight). Flowing venules (20–24 venules/mouse) in the chamber window were selected and mapped using intravital microscopy prior to hemin infusion. **(A)** Microvascular stasis was measured in the same venules 1 h after hemin infusion. **(B)** WBC counts were measured in blood 4 h after hemin infusion. Individual values are presented as circles with bars representing the means ± SEM; *p < 0.05 and ***p < 0.001, one-way ANOVA with Tukey’s multiple comparison test.

Since activation of NRF2-responsive genes has been shown to be beneficial in SCD mice, BACH1 inhibition was also evaluated. Townes HbSS mice were gavaged once daily, 6 days per week for 4 weeks with vehicle (VEH), ASP8731 (1, 3, 25 mg/kg body weight), hydroxyurea (HU, 100 mg/kg), or ASP8731 (25 mg/kg) + HU (100 mg/kg). On the last day of treatment, dorsal skinfold chambers were implanted on the mice. Flowing venules (20–23 venules/mouse) in the chamber window were selected and mapped using intravital microscopy. Mice were then infused with hemin (3.2 μmol/kg body weight) *via* the tail vein. One hour after hemin infusion, the venules were visually re-examined for stasis (no flow). The static venules in each mouse were counted and percent stasis at 1 h was calculated by dividing the number of static venules by the total (static + flowing) number of venules (Figure 2A). ASP8731 inhibited hemin-induced stasis in a dose responsive manner with the highest stasis occurring in HbSS mice pretreated with vehicle (33%) and decreasing to 26, 21, and 7% stasis in mice treated with 1, 3, and 25 mg/kg ASP8731, respectively. Treatment with HU (100 mg/kg) decreased stasis to 13%, with the lowest stasis (3.5%) seen in mice treated with ASP8731 (25 mg/kg) + HU (100 mg/kg).

### White blood cell counts in HbSS mice

After stasis measurements, blood samples were collected 4 h after the infusion of hemin. White blood cells (WBC) were counted manually in whole blood. WBC counts decreased in a manner similar to stasis (Figure 2B). ASP8731 significantly inhibited WBC counts in a dose-responsive manner with the highest WBC counts occurring in HbSS mice pretreated with vehicle (28 × 10^3^/μL) and decreasing to 22 × 10^3^/μL, 18 × 10^3^/μL, and 13 × 10^3^/μL in mice treated with 1, 3, and 25 mg/kg ASP8731, respectively. Treatment with HU (100 mg/kg) decreased the WBC count to 11 × 10^3^/μL. Treatment with ASP8731 (25 mg/kg) + HU (100 mg/kg) decreased the WBC count to 12 × 10^3^/μL.

### Red blood cell indices in HbSS mice

There were no significant differences in any of the red blood cell indices, including red blood cell counts, hemoglobin levels, hematocrits, and reticulocyte counts in any of the treatment groups as compared to vehicle treated HbSS mice (Supplementary Figure 1). In addition, the spleen weights as a percent of body weight were also not significantly different between treatment groups suggesting that BACH1 inhibition did not significantly inhibit hemolysis in HbSS Townes mice.

### Hepatic HO-1, NF-ĸB, ICAM-1, and BACH1 expression in HbSS mice

**Figure 3:**
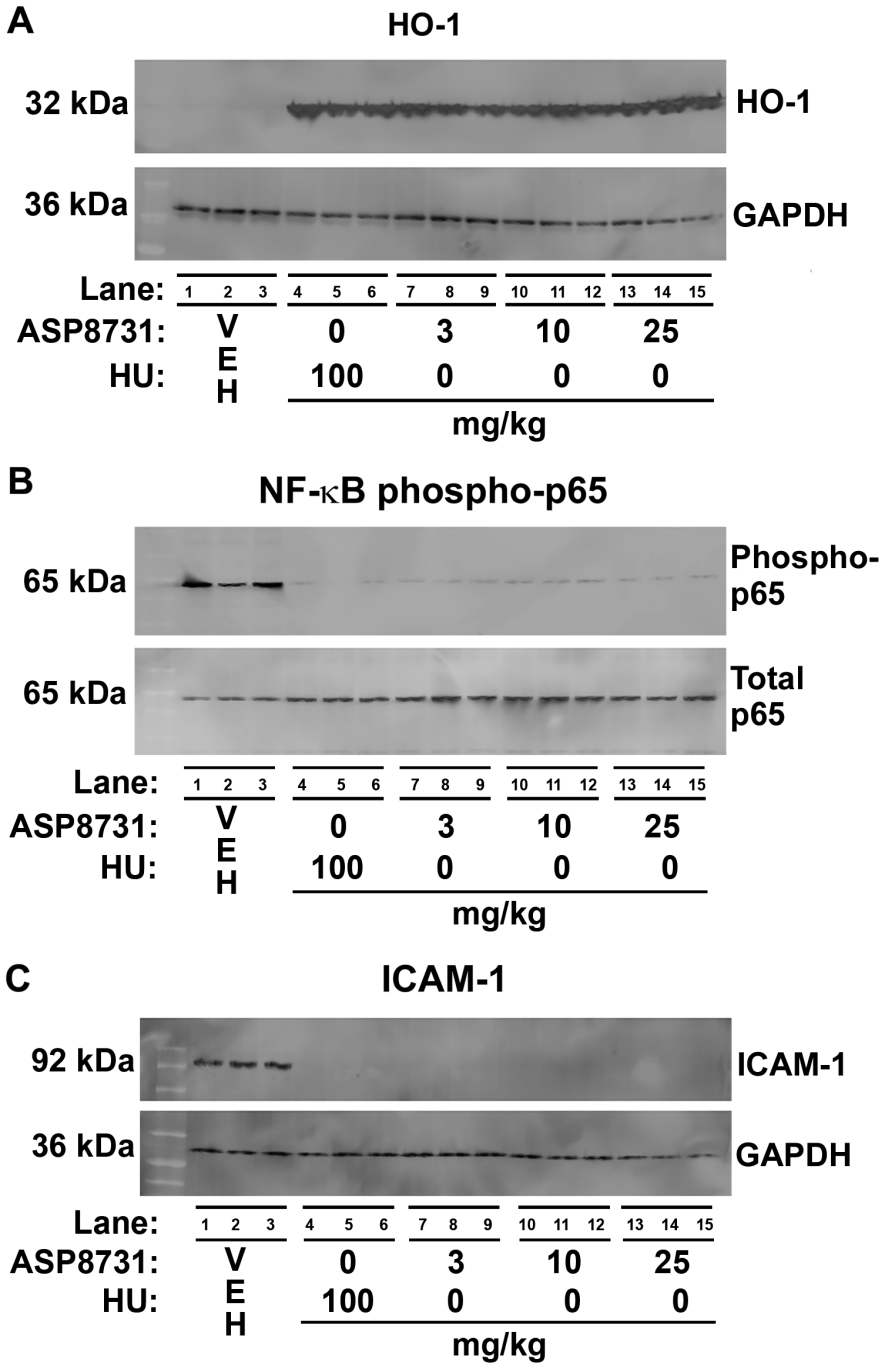
Bach1 inhibitor ASP8731 had anti-inflammatory properties in Townes HbSS mice. Townes HbSS mice (*n* = 3 mice/treatment, 2 males and 1 female) were gavaged once daily for 14 consecutive days with vehicle (VEH), ASP8731, or HU at the indicated doses. On the last day of treatment, mice were infused with hemin (3.2 μmol/kg). Livers were excised 4 h after the infusion of hemin. Proteins in liver were analyzed by Western blots. **(A)** HO-1 and GAPDH. **(B)** NF-ĸB phospho-and total p65. **(C)** ICAM-1 and GAPDH using liver microsomes **(A,C)** and nuclear extracts **(B)**.

In a separate cohort of HbSS mice, we examined the effects of ASP8731 and HU on HO-1, NF-ĸB phospho-p65 and ICAM-1 expression in the liver. HbSS mice (*n* = 3 mice/treatment, 2 males and 1 female) were gavaged once daily for 14 consecutive days with vehicle (VEH), ASP8731, or HU at the indicated doses. On the last day of treatment, mice were infused with hemin (3.2 μmol/kg) *via* the tail vein. Livers were collected 4 h after the infusion of hemin. Hepatic HO-1, NF-ĸB phospho-p65, and ICAM-1 proteins were analyzed on Western blots (Figures 3A–C) Relative expression of the bands on the Western blots were quantified using densitometry (Supplementary Figure 2). Microsomal HO-1 was significantly increased in the livers of HbSS mice treated with ASP8731 (25 mg/kg), HU (100 mg/kg), and ASP8731 (25 mg/kg) + HU (100 mg/kg). Nuclear NF-ĸB phospho-p65, a marker of NF-ĸB activation, was significantly decreased in the livers of HbSS mice at all treatment doses of ASP8731 and HU. Microsomal ICAM-1 was also significantly decreased in livers at all doses of ASP8731 and HU. Treatment of HbSS mice with ASP8731 increased nuclear BACH1 expression in the liver (Supplementary Figure 3). Similarly, HU also increased nuclear BACH1 protein expression.

### Hepatic heme oxygenase activity in HbSS mice

In a separate set of studies, ASP8731 dose responsively increased heme oxygenase (HO) activity in the livers of HbSS mice (Supplementary Figure 4). HU also increased HO activity to a somewhat lesser degree than ASP8731, but the highest HO activity was measured in mice treated with ASP8731 + HU.

### Gamma globin and HbF expression in HbSS mice

**Figure 4:**
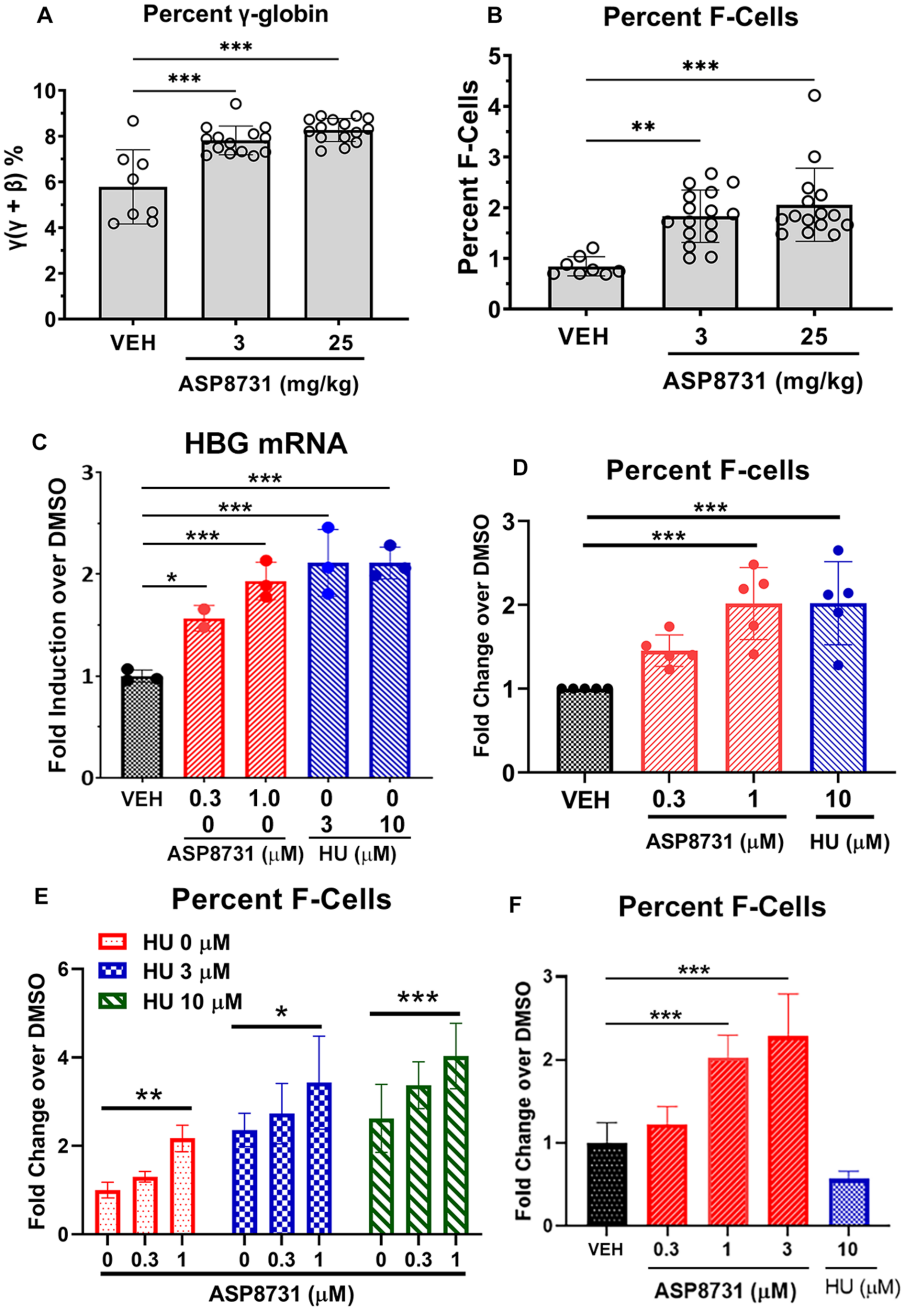
BACH1 inhibitor ASP8731 induces gamma globin and F-cells in HbSS mice and human erythroid differentiated CD34 cells. Townes HbSS mice (*n* = 8–16 mice/treatment, equal males and females) were gavaged once daily for 14 consecutive days with vehicle (VEH, *n* = 8 mice) or ASP8731 at 3 mg/kg (*n* = 16 mice) or 25 mg/kg (*n* = 16 mice). Whole blood was collected on the last day of treatment and the red blood cells (RBC) were lysed in water and membrane debris was moved by centrifugation and filtration. **(A)** Gamma (ɣ)-A and beta (β)-S globins in the lysed RBC supernatants were measured by reverse phase HPLC and expressed as percent gamma-globin (ɣ/ɣ + β). **(B)** HbF containing F-cells in whole blood were measured by FACS and expressed as percent F-cells [(HbF+ and Ter119+)/total RBC (Ter119+)]. **(C–F)** Human BM derived CD34+ cells were expanded (days 1–7) and differentiated (days 7–14) to erythroid cells in cell culture. Cells were differentiated in the presence of the indicated concentrations of ASP8731, HU, or DMSO (VEH) on days 7–14. Cells were collected for *HBG* mRNA or percent F-cell FACS analysis on day 14. **(C)** *HBG* mRNA was measured by Nanostring analysis (*n* = 3 subjects). **(D–F)** Percent F-cells were measured by FACS. Percent F-cells [(CD71+ and HbF+)/(CD71+)] was calculated and expressed as fold change over DMSO (VEH)-treated cells. **(D)** Values are mean ± SD of 5 subjects. **(E)** Values are the mean of 3 subjects measured in triplicate. **(F)** In one subject’s CD34 cells, HbF was not increased in response to HU, but these cells were responsive to ASP8731. Mean ± SD of triplicate measurements from one donor. *p < 0.05, **p < 0.01, and ***p < 0.001, one-way ANOVA with Tukey’s multiple comparison test.

BACH1 inhibitor ASP87311 was examined for its ability to increase expression of gamma globins and HbF-containing F-cells. Gamma and beta-S globin levels were measured by reverse phase HPLC in the red blood cells of Townes HbSS mice after treatment with ASP8731 (3 or 25 mg/kg) or vehicle once daily for 14 days. The ratio of gamma globin to gamma globin + beta-S globin increased from a mean of 5.8% in vehicle treated HbSS mice to 7.8 and 8.3% in HbSS mice treated with 3 and 25 mg/kg ASP8731, respectively (Figure 4A). Similarly, HbF containing F-cells measured by FACS significantly increased from 1.0% of red blood cells in vehicle-treated HbSS mice to 1.8 and 2.1% in HbSS mice treated with 3 and 25 mg/kg ASP8731, respectively (Figure 4B).

### HGB mRNA and F-cells in human erythroid differentiated CD34 cells

The effects of BACH1 inhibitor ASP8731 and HU on gamma globin gene (*HBG*) mRNA levels and F-cell production were measured in human BM-derived CD34 cells during erythroid differentiation. HGB mRNA was significantly increased in CD34 cells differentiated in the presence of ASP8731 at 0.3 and 1 μM or in the presence of HU at 3 and 10 μM (Figure 4C) as compared to the DMSO vehicle (VEH). The percent F-cells was significantly increased in CD34 cells differentiated in 1 μM ASP8731 or 10 μM HU (Figure 4D; Supplementary Table 2). Differentiation of cells to CD71+ cells (reticulocyte marker) was not affected by treatment with HU or ASP8731 (Supplementary Table 2). In preliminary time course study, incubation of compounds for different time (days, 3, 7, and 10) did not change the % CD71+ cells (data not shown). F-cells were increased by both ASP8731 and HU individually, but the combination of both compounds increased F-cells higher than either drug alone (Figure 4E). In one healthy CD34 donor’s cells that were non-responsive to HU, ASP8731 was able to induce F-cells at 1 and 3 μM (Figure 4F).

### Gamma-, alpha-, and beta-globin in erythroid differentiated CD34 cells derived from SCD subjects

**Figure 5:**
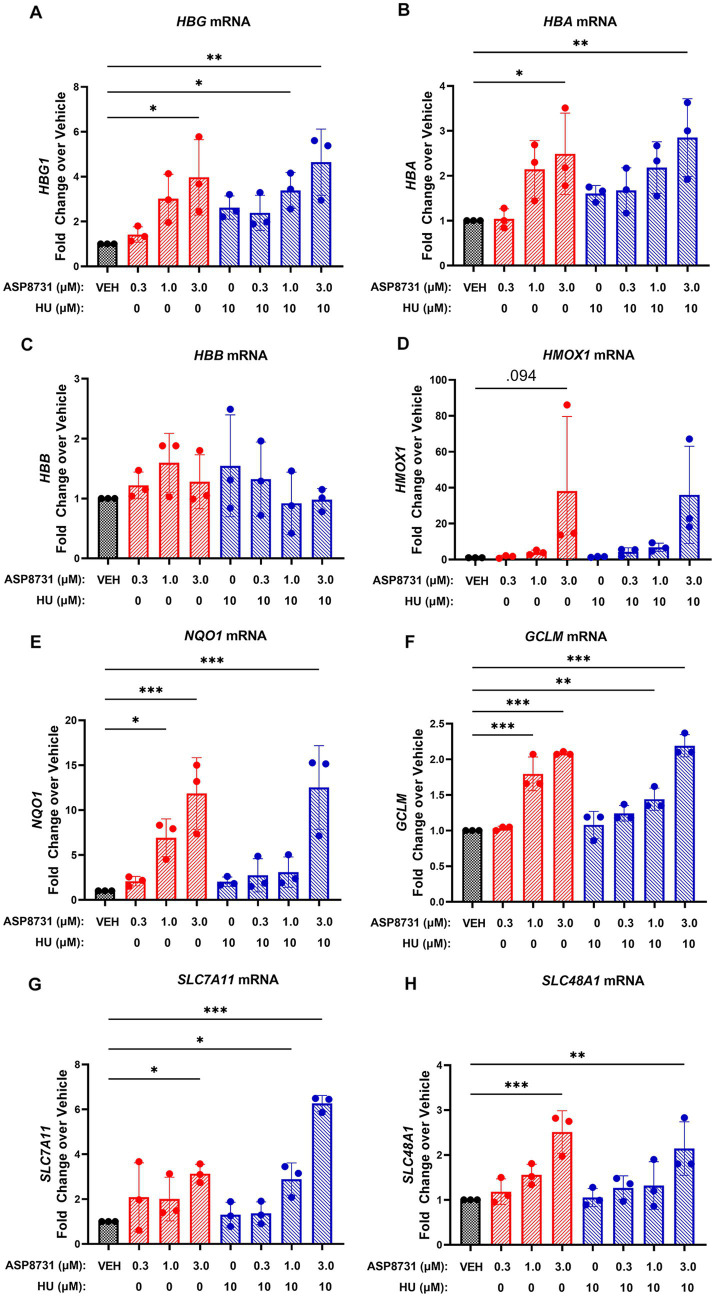
BACH1 inhibitor ASP8731 induces gamma-and alpha-globin, but not beta-globin in erythroid differentiated CD34 cells derived from SCD subjects. Peripheral blood CD34+ cells isolated from SCD subjects (*n* = 3) were expanded (days 1–7) and differentiated (days 7–10) to erythroid cells in cell culture. Cells were differentiated in the presence of the indicated concentrations of ASP8731, HU, or DMSO (VEH) on days 7–10. Cells were collected for mRNA analysis by Nanostring on day 10. **(A)** *HBG* mRNA; **(B)** *HBA* mRNA; **(C)** *HBB* mRNA – the probes used for Nanostring for *HBB* do not distinguish between normal and sickle; **(D)** *HMOX1* mRNA; **(E)** *NQO1* mRNA; **(F)** *GCLM* mRNA; **(G)** *SLC7A11* mRNA; and **(H)** *SLC48A11* mRNA. Values are expressed as fold change over DMSO vehicle and bars are mean ± SD. *p < 0.05, **p < 0.01, and ***p < 0.001 compared to vehicle, one-way ANOVA followed by Dunnett’s multiple comparison test.

The effects of BACH1 inhibitor ASP8731 and HU were examined in erythroid differentiated CD34 cells derived from the peripheral blood of SCD subjects (Figure 5). Levels of (A) gamma globin (*HBG*), (B) alpha globin (*HBA*), (C) beta globin (*HBB*), (D) HO-1 (*HMOX1*), (E) NAD(P)H quinone dehydrogenase 1 (*NQO1*), (F) glutamate-cysteine ligase modifier subunit (*GCLM*), (G) solute carrier family 7 member 11 (*SLC7A11*), and (H) solute carrier family 48 member 1 (*SLC48A1*) mRNA levels were measured using Nanostring. ASP8731 (10 μM) and the combination of ASP8731 (10 μM) + HU (10 μM) significantly increased the mRNA of every gene tested except *HBB* and *HMOX1*. The lack of statistical difference in HMOX1 gene was primarily due to very high level of expression in 1 patient sample, thus fold change at 3, 1, and 0.3 μM ASP8731 and 10 μM HU were 38.16 ± 41.5; 3.96 ± 1.28, 1.55 ± 0.8, and 1.51 ± 0.3, respectively. In contrast HU alone at 10 μM concentration did not significantly increase the mRNA of any of the genes tested. These data suggest that the BACH1 inhibitor ASP8731 is a potent inducer of HbF and antioxidant response genes in SCD patients.

## Discussion

BACH1 inhibitor ASP8731 potently modulated multiple pathways that affect SCD pathophysiology. In HepG2 liver cells, ASP8731 increased *HMOX1* and *FTH1* mRNA. In pulmonary endothelial cells, ASP8731 decreased *VCAM1* mRNA in response to TNF-α and prevented a decrease in glutathione in response to hemin. BACH1 has been reported to repress transcription of genes that reduce labile iron, oxidative stress, and ferroptosis (22).

Both ASP8731 and HU markedly inhibited heme-mediated microvascular stasis in a dorsal skin-fold chamber model in HbSS mice while increasing hepatic HO-1 expression. HO-1 induction might have had a large impact on the inhibition of microvascular stasis in HbSS mice in part due to the many anti-inflammatory effects of HO-1 (11, 12). Markers of inflammation including ICAM-1 and NF-kB phospho-p65 expression in the liver and blood WBC counts were significantly decreased by ASP8731 treatment as compared to vehicle controls. Inhibition of HO-1 activity with tin protoporphyrin has been previously shown to abrogate the beneficial effects of NRF2 activation on microvascular stasis (15). The potent anti-inflammatory effects of HO-1 are related in part to the release of carbon monoxide and biliverdin and the post-transcriptional induction of ferritin heavy chain (*FTH1*) by iron generated by HO-1-mediated heme degradation (11, 23, 24).

Paradoxically, treatment of HbSS mice with ASP8731 increased nuclear BACH1 expression in the liver, suggesting a there is a feedback loop that increases nuclear BACH1 protein expression upon inhibition of BACH1. Similarly, HU also increased nuclear BACH1 protein expression. HU is a known nitric oxide (NO) donor (25). The ability of NO alone or in combination with heme to induce Nrf2/HO-1 has been reported (26).

Microvascular stasis was measured in the subcutaneous venules in the skin, but we did not directly measure inflammatory markers in the skin. However, it has previously been shown that inflammation in HbSS mice is occurring in blood vessels throughout the vasculature including the skin, liver, kidneys, and lungs (3, 11, 15, 27). We measured hepatic HO-1 expression and activity in HbSS mice, but not in other organs. We previously showed that genetic overexpression of hepatic HO-1 has systemic effects on microvascular stasis in the distal skin (12). It is likely that ASP8731 had anti-inflammatory effects in all tissues as evidenced by the significant reduction in the WBC counts.

HU induced HO-1 to a similar degree as ASP8731 in HbSS mice. To our knowledge, this is the first time HU has been reported to increase HO-1 expression. Lanaro and colleagues (28) reported increased *HMOX1* mRNA in the mononuclear cells and neutrophils of SCD patients. In that study, SCD patients taking HU had a trend toward higher *HMOX1* mRNA than SCD patients not on HU, but this difference did not appear to be significant. HU increases nitric oxide (NO) production, acting as both a NO donor and a stimulator of NO synthase (25, 29). Bioactivation of NO is a plausible mechanism for *HMOX1* induction by HU as NO can activate KEAP1/NRF2 signaling (30).

Clinical studies have shown that increasing HbF levels with HU inhibits the polymerization of HbS, decreases the severity of many clinical features of SCD, and improves survival (31–33). SCD patients with hereditary persistence of F-cells have a more benign clinical course (34). Given the modest increase in F-cells in HbSS mice treated with ASP8731 (0.8% F-cells in vehicle treated mice compared to 1.8 and 2.1% in mice treated with 3 mg/kg and 25 mg/kg of ASP8731, respectively), F-cell induction is probably not a major factor in the anti-vaso-occlusion protection seen in HbSS mice. Humans at birth express 2 gamma-globin genes (*HBG1* and *HBG2*). *HBG1* codes for A-gamma (^A^ɣ)-globin and *HBG2* codes for G-gamma (^G^ɣ)-globin. The 2 globin chains differ by one amino acid, with (^A^ɣ) having an alanine and (^G^ɣ) having a glycine at position 136. Two gamma globin chains with two alpha (α)-globin chains form HbF (α_2_/ɣ_2_), which is normally replaced by HbA shortly after birth. Townes HbSS mice have the human *HBG1* gene, but not the human *HBG2* gene, and thus they can only express ^A^ɣ-globin. Murine *HBB* genes in the Townes model were replaced by human *HBG1* and *HBBS* genes and some proximal but not some distal gene-regulatory elements. The HbSS Townes humanized knock-in mouse model recapitulates human globin gene switching (35). HU suppresses erythropoiesis in Townes HbSS mice to create an early persistent F-cell phenotype without re-activating γ-globin transcription (35). Townes mouse HSCs, in contrast to human HSCs, fail to induce HbF to therapeutic levels after genetic disruption of the BCL11A binding site in the *HBG1* promoter (36). This is likely due to missing distal regulatory elements of the globin genes in the Townes model (36). Thus, interpretation of HbF induction in Townes HbSS mice has limitations.

BACH1 and BACH2 support erythropoiesis by regulating heme metabolism in committed erythroid cells, driving cells toward erythroid differentiation and away from myeloid differentiation (37) by repressing C/EBPβ, a transcription factor regulating myelopoiesis and inflammation. Heme-BACH1 interaction also plays an important role in globin gene expression. The binding of heme to CP motifs in BACH1 inhibits BACH1 repressor activity. The human globin gene cluster spans ~70 kb and contains 5 globin genes including ϵ, γ_G_, γ_A_, δ, and β that are controlled by the microlocus control region (μLCR) (38). BACH1 forms heterodimers with small Maf proteins that repress globin expression by binding to Maf recognition elements (MARE) in the μLCR (9). Heme increases globin gene expression by binding to BACH1 and blocking the interaction of BACH1 with MARE regions in the μLCR (38, 39). A role for BACH1 in the control of gamma globin expression has been suggested, but not shown directly (40, 41). Our studies indicate that BACH1 inhibition by ASP8731 induces gamma-globin and HbF expression in human erythroid cells.

In BM derived CD34+ cells from normal subjects, BACH1 inhibitor induced *HBG* mRNA and percent F-cells. ASP8731 and heme alleviate BACH1 repression, leading to activation of NRF2-responsive genes. Activation of NRF2 promotes ^A^ɣ- and ^G^ɣ-globin expression in erythroid cells (42–46), which increase HbF expression in red blood cells of SCD patients. Interestingly, ASP8731 + HU potentiated the induction of F-cells over HU alone. In one donor’s CD34 cells that were non-responsive to HU, ASP8731 was able to induce F-cells. The effectiveness of HU to induce HbF is variable in SCD patients and there are concerns over the long-term efficacy of HU (31, 47–49). Induction of F-cells in an HU non-responder by BACH1 inhibition, albeit a single subject, suggests this therapeutic might be an option for patients who are not responsive to HU. During differentiation of CD34+ cells from SCD patients, BACH1 inhibition increased the expression of *HBG*, *HBA* and other NRF2 responsive genes.

In conclusion, BACH1 inhibitors are promising therapeutics for SCD that enhance the cytoprotective oxidative stress responses and thereby reduce inflammation and vaso-occlusion. BACH1 inhibitors have the added benefit of inducing HbF expression and thereby potentially reducing HbS polymerization and hemolysis. The present data support evaluation of BACH1 inhibitors as therapeutics for SCD patients.

## Data availability statement

The raw data supporting the conclusions of this article will be made available by the authors, without undue reservation.

## Ethics statement

The animal study was reviewed and approved by University of Minnesota’s Institutional Animal Care and Use Committee.

## Author contributions

JB wrote the manuscript, analyzed data, oversaw the studies in mice, and prepared figures. FA, CC, PZ, JN, and CR conducted mouse experiments and data collection, processing, and analysis. GV analyzed data and edited the manuscript. JS, LO, EC, and YH planned and conducted cell culture experiments, data collection, processing, and analysis, and prepared figures. SD, MS, MB, DS, and SN: conception, study design and data interpretation. All authors contributed to the article and approved the submitted version.

## Funding

This study was funded by Astellas Pharma Inc. and investigators received funding to conduct this study from the study sponsor.

## Conflict of interest

JB and GV are consultants and receive research funding from Astellas Pharma/Mitobridge. SN, SD, LO, and JS are employees of Astellas Pharma US Inc. MS, DS, and MB were employees of Astellas Pharma US Inc./Mitobridge at the time the study was conducted. EC and YH were employed by company ReachBio.

The remaining authors declare that the research was conducted in the absence of any commercial or financial relationships that could be construed as a potential conflict of interest.

The authors declare that this study received funding from Astellas-Mitobridge. The funder had the following involvement: study design, data collection, analysis, interpretation of data, and the writing of this article.

## Publisher’s note

All claims expressed in this article are solely those of the authors and do not necessarily represent those of their affiliated organizations, or those of the publisher, the editors and the reviewers. Any product that may be evaluated in this article, or claim that may be made by its manufacturer, is not guaranteed or endorsed by the publisher.
