# Supplementary material for: The BACH1 inhibitor ASP8731 inhibits inflammation and vaso-occlusion and induces fetal hemoglobin in sickle cell disease
Source: Front Med (Lausanne). 2023 Apr 18;10:1101501. doi: 10.3389/fmed.2023.1101501 (PMC10152901; doi:10.3389/fmed.2023.1101501)
Supplement: Supplementary file 1 [file Data_Sheet_1.PDF]

SUPPLEMENTAL MATERIAL

The BACH1 Inhibitor ASP8731 Inhibits Inflammation and Vaso-occlusion and Induces Fetal Hemoglobin in Sickle Cell Disease

John D. Belcher[1], Selvaraj Nataraja[2], Fuad Abdulla[1], Ping Zhang[1], Chunsheng Chen[1], Julia Nguyen[1], Conglin Ruan[1], Maneet Singh[2], Shilpa Demes[3], Lyndsay Olson[2], Domi Stickens[2#], Jeff Stanwix[2], Emer Clark[4], Yongzhao Huang[4], Margaret Biddle[2*], Gregory M. Vercellotti[1],

[1]University of Minnesota, Minneapolis, MN; [2]Mitobridge Inc., Cambridge, MA; [3]Astellas Pharma Global Development Inc, Northbrook, IL; [4]ReachBio, Seattle, WA

Present affiliations: *- Rheos Medicine, Cambridge, MA; #- D3A Biopharma Consulting, Boston, MA

SUPPLEMENTAL MATERIAL

MATERIALS AND METHODS

Western blot densitometry and quantification

Relative band intensities and backgrounds on immunoblot images were measured using ImageJ software (NIH). Integrated band densities were expressed relative to the appropriate loading control after background subtraction.

Heme oxygenase (HO) enzyme activity

Heme oxygenase (HO) activity was measured as previously described [1] in liver microsomes. Microsomes (2mg) in 2mM MgCl2, 0.1M K2HPO4 buffer, pH 7.4 were added to the reaction mixture (400 µl, final volume) containing 2.5 µg of recombinant biliverdin reductase (Enzo Life Sciences), 2 mM glucose-6-phosphate (Sigma-Aldrich), 0.2U glucose-6-phosphate dehydrogenase (Sigma-Aldrich), 50 µM hemin chloride (Frontier Scientific Porphyrin Products) and 0.8mM NADPH (Sigma-Aldrich) for 1 h in the dark. Bilirubin that was formed was extracted into chloroform (Sigma-Aldrich) and measured by the delta O.D. at 464-530 nm (extinction coefficient, 40 mM-1 cm-1 for bilirubin). HO activity is expressed as pmol of bilirubin formed/mg microsomal protein/h.

Supplemental Table 1.

NanoString Probe Sequences

Gene	Probe	Sequence (5' → 3')
GCLM	Probe A	ACTCCAAGGACTGAACAGGCCATGTCAACTGCACTTCTAGTTGATGATGACCTGCCAATGCACTCGATCTTGTCATTTTTTTGCG
	Probe B	CGAAAGCCATGACCTCCGATCACTCCTCCATCTTCAATAGGAGGTGAAGCAATGATCACAGAATCCAGCTGTGCA
HBG	Probe A	GTTCTCAGGATCCACATGCAGCTTGTCACAGTGCAGCCTGGAGTTTATGTATTGCCAACGAGTTTGTCTTT
	Probe B	CGAAAGCCATGACCTCCGATCACTCCCGAAATGGATTGCCAAAACGGTCACCAGCACATTTCCCAGGAGCTTGAA
HBA1	Probe A	CAGCGCGTTGGGCATGTCGTCCACGTGCGCCACGGCAGATAAGGTTGTTATTGTGGAGGATGTTACTACA
	Probe B	CGAAAGCCATGACCTCCGATCACTCCGAAGCTTGTGCGCGTGCAGGTCGCTCAGGGCGGA
HBB	Probe A	AATTGGACAGCAAGAAAGCGAGCTTAGTGATACTTGTGGGCCAGGGCATTCTTCCTTCCTGTGTTCCAGCTACAAACTTAGAAAC
	Probe B	CGAAAGCCATGACCTCCGATCACTCATCCCCCAGTTTAGTAGTTGGACTTAGGGAACAAAGGAACCTTTAATAGA

HMOX1	Probe A	CCACCCACGCATGGCTCAAAAACCACCCCAACCCTGCTATAAAAACAACACAAACTGGAGAGAGAAGTGAAGACGATTTAACCCA
	Probe B	CGAAAGCCATGACCTCCGATCACTCTTCACACAAAAGTTAGACCAAGGCCACAGTGCCGTTAAACACCTCCCTCC
NQO1	Probe A	CCCAAATATTCTCCAGGCGTTTCTTCCATCCTTCCAGGATTTGAATTCGGCCAGCAGACCTGCAATATCAAAGTTATAAGCGCGT
	Probe B	CGAAAGCCATGACCTCCGATCACTCGAAGTTTAGGTCAAAGAGGCTGCTTGGAGCAAAATACAGTGGTGTCTCAT
SLC48A1	Probe A	GGTGGGCATAGAGGCTGAGCAGGAAGGCCCACTTGAAGGAAATGAAGCCTTTCGTTGGGACGCTTGAAGCGCAAGTAGAAAAC
	Probe B	CGAAAGCCATGACCTCCGATCACTCCAGAAATCGCTGAGGATGCTGATGTCAGCAAAGTCAGCCCGGTAGC
SLC7A11	Probe A	GGCGTATTATGAGGAGTTCCACCCAGACTCGTACAAAAGCTGGTAATGGACTGGTCAAGACTTGCATGAGGACCCGCAAATTCCT
	Probe B	CGAAAGCCATGACCTCCGATCACTCTTCCAGAATGTAGCGTCCAAATGCCAGGGATATCACAGCAGTAGCTGCAG

Housekeeping Reference Genes - NanoString Probe Sequences

Gene	Probe	Sequence (5' → 3')
CLTC	Probe A	CAGACTCCATAGTCAGGGTACTGAAGCCAATGTTTGCTGGGTTGATACCCCTGAGGCTGTTAAAGCTGTAGCAACTCTTCCACGA
	Probe B	CGAAAGCCATGACCTCCGATCACTCTACCACCTGGGCCTGCTCTCCTACTTTTTCTCTAATGCAGATGAATTTGT
HPRT	Probe A	TGAGCACACAGAGGGCTACAATGTGATGGCCTCCCATCTCCTTCATCACACATTTGGAATGATGTGTACTGGGAATAAGACGACG
	Probe B	CGAAAGCCATGACCTCCGATCACTCCAGTGCTTTGATGTAATCCAGCAGGTCAGCAAAGAATTTATAGCCCCCCT
PPIA	Probe A	ACCCGTATGCTTTAGGATGAAGTTCTCATCTTCAAATTTCTCCCCATAGACCACGCGATGACGTTCGTCAAGAGTCGCATAATCT
	Probe B	CGAAAGCCATGACCTCCGATCACTCGAACCATTTGTGTTGGGTCCAGCATTTGCCATGGACAAGATGCCAGG

Supplemental Table 2.

F-cells (CD71+/HbF+) as % of Parent-cells		
	Mean	SD
ASP8731 (1 μM)	12.42%	0.05
ASP8731 (0.3 μM)	9.73%	0.06
HU (10 μM)	12.95%	0.08
DMSO (0.1%)	6.20%	0.03

Reticulocytes (CD71+ bright) as % Parent Cells		
	Mean	SD
ASP8731 (1 μM)	31.72%	0.03
ASP8731 (0.3 μM)	36.20%	0.12
HU (10 μM)	28.55%	0.14
DMSO (0.1%)	34.53%	0.11

F-cells (CD71+/HbF+) and reticulocytes (CD71+ bright) expressed as a percentage of parental cells measured by FACS in Figure 4D. Values are means and SD of 5 subjects.

SUPPLEMENTAL FIGURES

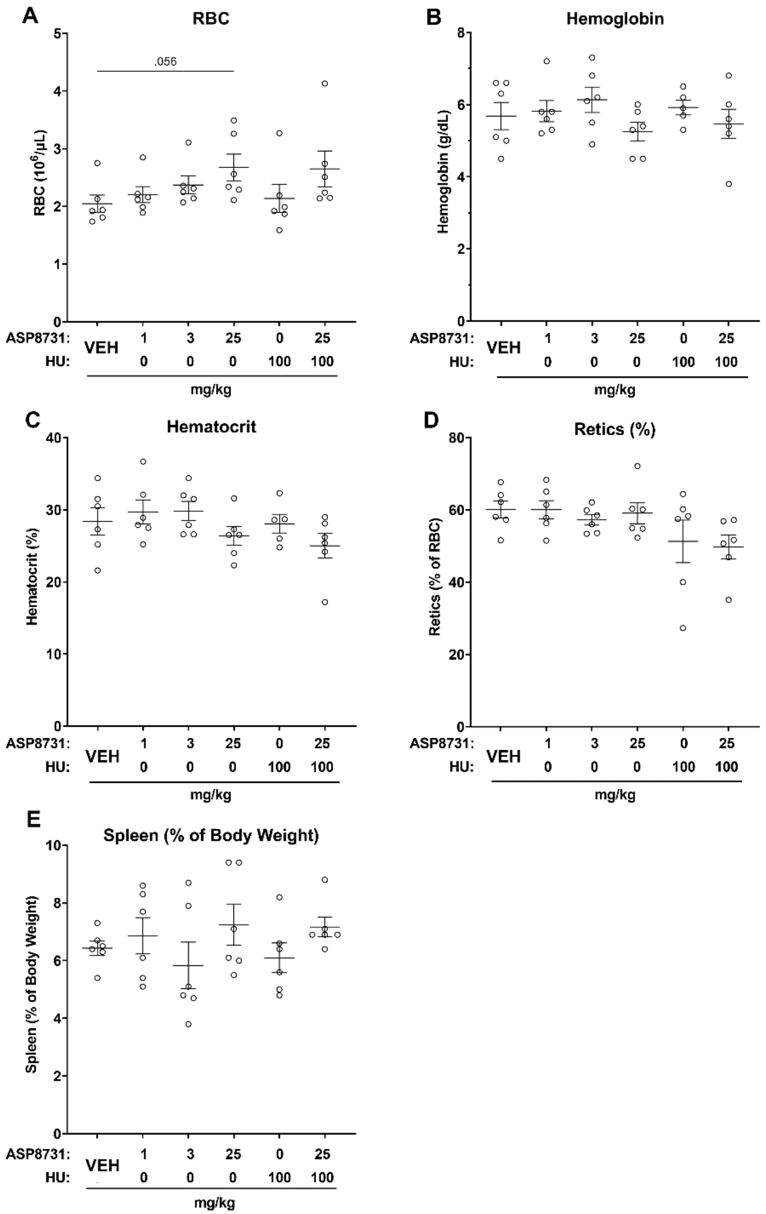

Supplemental Figure 1. Red blood cell indices and spleen weights did not change in HbSS mice treated with ASP871 or HU. Townes HbSS mice (n=6/group, 3 males and 3 females) were gavaged once daily with vehicle (VEH), ASP8731, or HU at the indicated doses for 4 weeks. (A) Red blood cells, (B) hemoglobin, (C) hematocrits, (D) percent reticulocytes (retics), and (E) spleen weights were measured after 4 weeks of treatment. (Kruskal-Wallis, with Dunn's multiple comparison test).

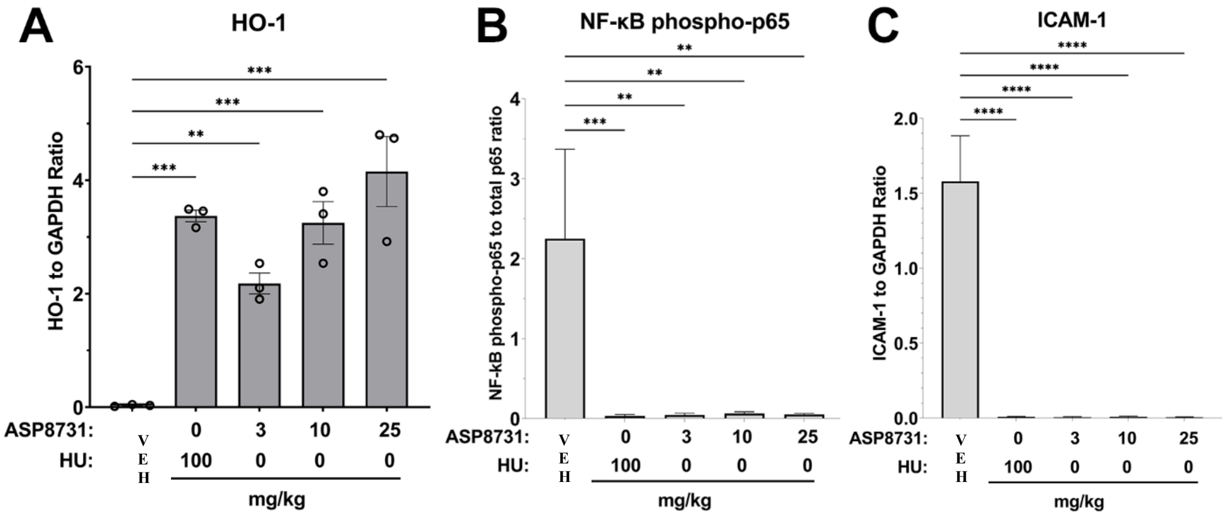

Supplemental Figure 2. Bach1 inhibitor ASP8731 increased anti-inflammatory HO-1 and decreased pro-inflammatory NF-κB phospho-p65 and ICAM-1 in the livers of hemin-treated Townes HbSS mice. HbSS mice (n=3/group, 2 males and 1 females) were gavaged once daily with vehicle (VEH), ASP8731, or HU at the indicated doses for 14 consecutive days. On the last day of treatment, mice were infused with hemin (3.2 µmol/kg). Livers were collected 4 h after the infusion of hemin. Liver microsomes (A and C) and nuclear extracts (B) were analyzed using Western blots (see Figure 3) and quantified. (A) HO-1 to GAPDH ratios are expressed relative to the mean ratio in vehicle (VEH)-treated mice; (B) NF-κB phospho-p65 to total p65 ratios; and (C) ICAM-1 to GAPDH ratios. Values are means ± SEM. **$P<0.01$, ***$p<0.001$, and ****$p<0.0001$; one-way ANOVA with Dunnett's multiple comparisons test.

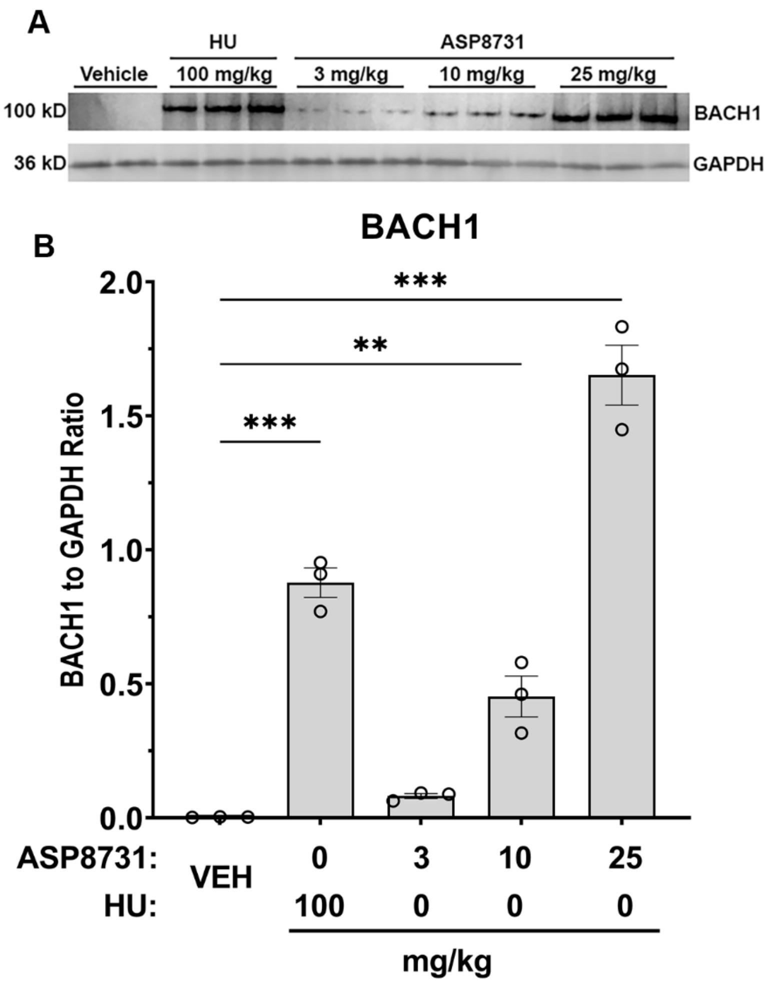

Supplemental Figure 3. Bach1 inhibitor ASP8731 increased nuclear BACH1 expression in the livers of hemin-treated Townes HbSS mice. HbSS mice (n=3/group, 2 males and 1 female) were gavaged once daily with vehicle (VEH), ASP8731, or HU at the indicated doses for 14 consecutive days. On the last day of treatment, mice were infused with hemin (3.2 μmol/kg). Livers were collected 4 h after the infusion of hemin. (A) BACH1 protein expression was analyzed using liver nuclear extracts by Western blot. (B) BACH1 bands were quantified by densitometry as a ratio relative to GAPDH loading control. Values are means ± SEM of BACH1. **P<0.01 and ***p<0.001, one-way ANOVA with Dunnett's multiple comparisons test.

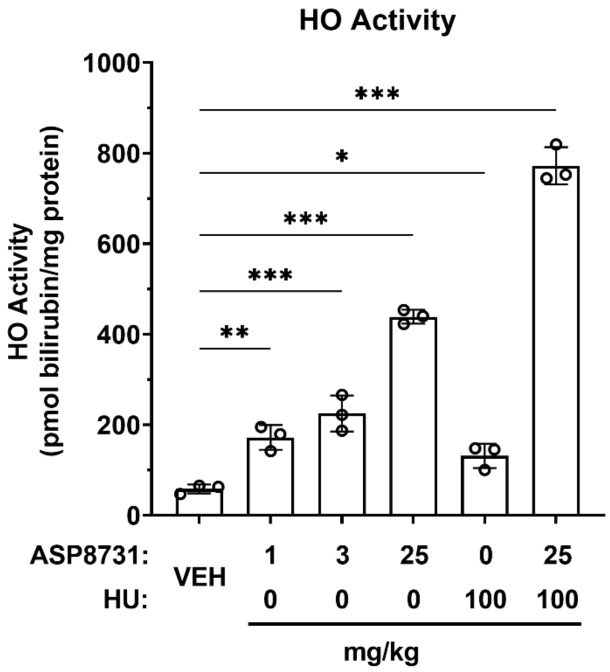

Supplemental Figure 4. Bach1 inhibitor ASP8731 increased heme oxygenase (HO) activity in the livers of Townes HbSS mice. HbSS mice (n=3/group, 2 males and 1 female) were gavaged once daily with vehicle (VEH), ASP8731, HU, or ASP8731 + HU at the indicated doses for 4 weeks. Livers were collected on the last day of treatment. Microsomes were isolated from each liver and hepatic HO activity was measured and expressed as bilirubin production. Values are means ± SEM. *P<0.05, **p<0.01, and ***p<0.001, one-way ANOVA with Dunnett's multiple comparisons test.

SUPPLEMENTAL REFERENCE

1. Balla, G., et al., *Ferritin: a cytoprotective antioxidant strategem of endothelium.* J Biol Chem, 1992. **267**(25): p. 18148-53.
